# Intraluminal catheter plasty. An alternative technique to remove tethered abdominal surgical drains

**DOI:** 10.1259/bjrcr.20210025

**Published:** 2021-04-29

**Authors:** Virjen Patel, Benedict Thomson, Narayanan Thulasidasan, Athanasios Diamantopoulos

**Affiliations:** 1 Department of Interventional Radiology, Guy’s and St. Thomas' NHS Foundation Trust, St Thomas' Hospital, London, United Kingdom; 2 School of Biomedical Engineering & Imaging Sciences, Faculty of Life Sciences & Medicine, Kings College London, London, United Kingdom

## Abstract

**Objective:**

Retained surgical drains can lead to significant complications including gastrointestinal fistulae, abscess formation and intestinal obstruction. Today, there is little in the literature describing the role of Interventional Radiology (IR) in assisting the removal of surgical drains. We describe the use of the well-established intraluminal catheter plasty technique, previously used for the removal of adhered central venous catheters, in order to remove a tethered abdominal drain.

**Methods and materials:**

A 67-year-old female was referred to the IR department for the removal of a 24 Fr Robinson’s intra-abdominal surgical drain that could not be removed by conventional methods, as there was a concern that it was kinked internally. Both fluoroscopy and cone beam CT were performed, which identified the drain to be sited within the pelvis with no kinks.

A first attempt to remove the drain over a 0.035 stiff wire was unsuccessful due to resistance and patient discomfort. We suspected that this was due to adhesions surrounding the drain. Consequently, a 9 × 40 mm angioplasty balloon was used over the wire with serial dilatations along the drain to disrupt the adhesions. Several areas of waisting were identified. The drain was gently withdrawn over the wire with minimal resistance. *Ex-vivo* inspection of the drain showed no evidence of structural damage and fluoroscopic imaging confirmed no retained fragments.

**Conclusion:**

We describe a safe and effective novel technique of intraluminal catheter plasty used to remove a tethered surgical drain following failed conventional methods.

## Introduction

Tethered surgical drains are an infrequent but challenging post-operative complication. Techniques employed to resolve this include applying traction, twisting and even cutting the drain followed by surgical retrieval under general anaesthesia.^1^ This can lead to an increase in morbidity and prolonged recovery time with extended length of stay. Retained drains can lead to significant complications such as gastrointestinal fistulae, abscess formation and intestinal obstruction.^2^

Intraluminal catheter plasty for tunnelled vascular line removal is a well-recognised technique performed by Interventional Radiologists.^3^ There is little in the literature of the role of Interventional Radiology (IR) in assisting to remove surgical drains. We describe a consolidated endovascular technique adapted for abdominal catheters, to successfully remove a tethered abdominal surgical drain using intraluminal catheter plasty.

## Case presentation

A 67-year-old female had previously undergone a laparotomy for excision of a para-aortic pelvic mass. On histological analysis, the mass was found to be metastatic squamous cell carcinoma of unknown origin. This was complicated by acute mesenteric ischaemia requiring a second laparotomy and a Hartman’s procedure. At the time of the second procedure, a 24 Fr Robinson’s surgical drain was inserted into the right iliac fossa.

The clinical team attempted to remove the surgical drain once there was minimal drain output, but were unsuccessful. The drain was felt to be tethered, causing the patient a significant amount of pain and discomfort. No clear cause for the tethered drain was identified. In order to avoid an additional surgical procedure, the case was discussed with the IR department. A decision was made to attempt drain removal over a stiff wire under fluoroscopic guidance, due to concerns of the drain being kinked internally.

## Treatment

**Figure 1.**
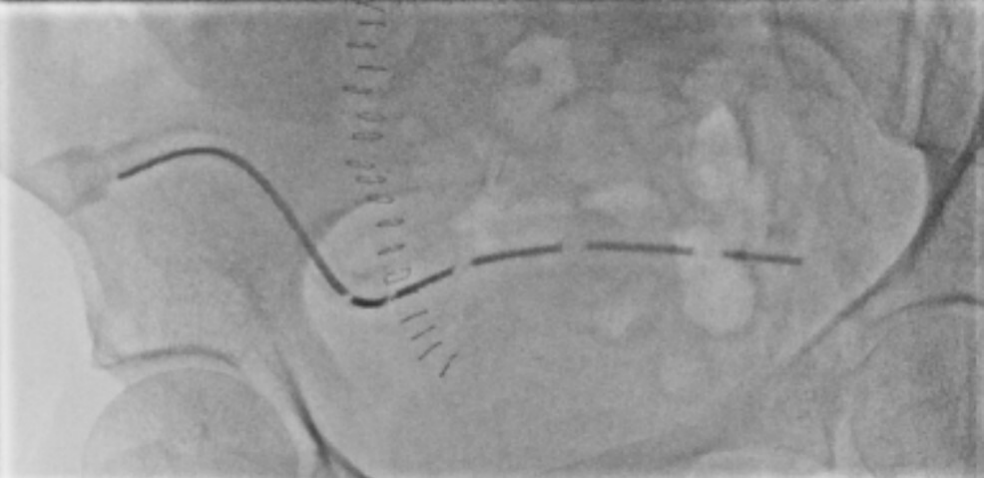
Initial fluoroscopic image of the right iliac fossa large bore surgical drain

**Figure 2.**
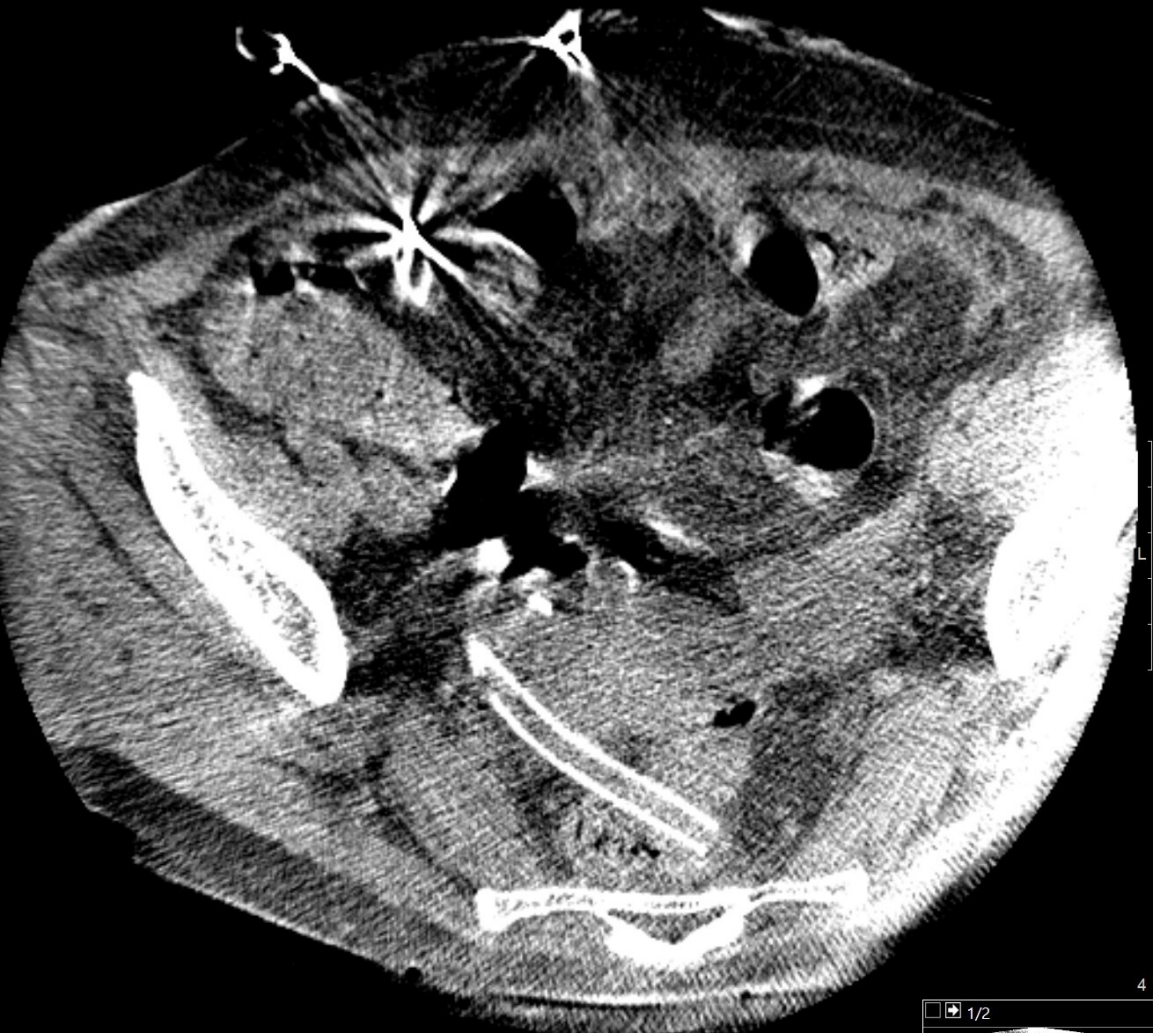
Cone beam CT identifying drain position and no evidence of kinking

The procedure was performed in the IR suite under conscious sedation. A large bore surgical drain was identified within the right iliac fossa. Initial fluoroscopic images did not demonstrate any kinks within the drain (Figure 1). A 180 cm 0.035-inch stiff Terumo wire (Terumo, Tokyo, Japan) was advanced into the drain. Attempted removal of the drain over the wire was unsuccessful due to significant resistance. A cone beam CT was performed for further investigation. The drain was sited within the pelvis with the tip positioned anterior to the sacrum, and no kinking of the tube was demonstrated (Figure 2). At that point, it was suspected that the drain was fixed due to significant adhesions.

**Figure 3.**
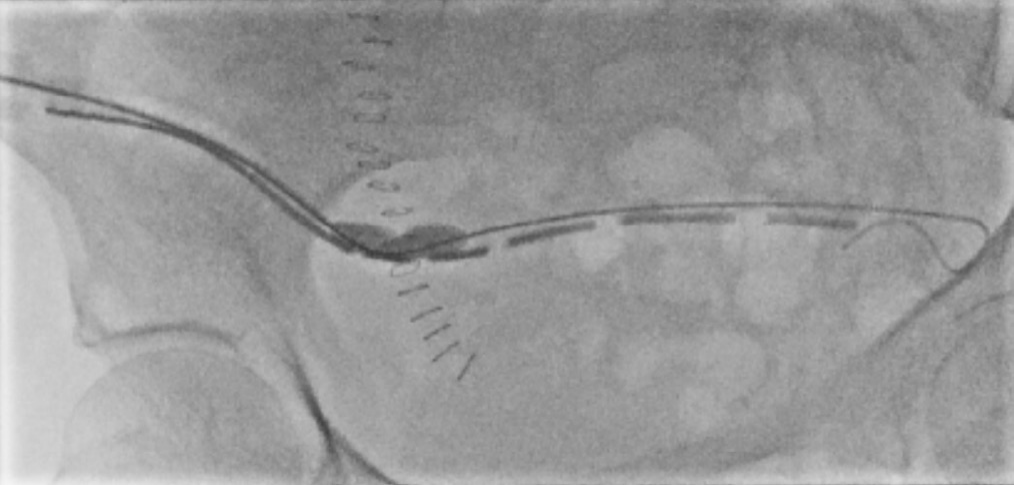
Intraluminal dilation using a vascular charger balloon demonstrates luminal waisting

**Figure 4.**
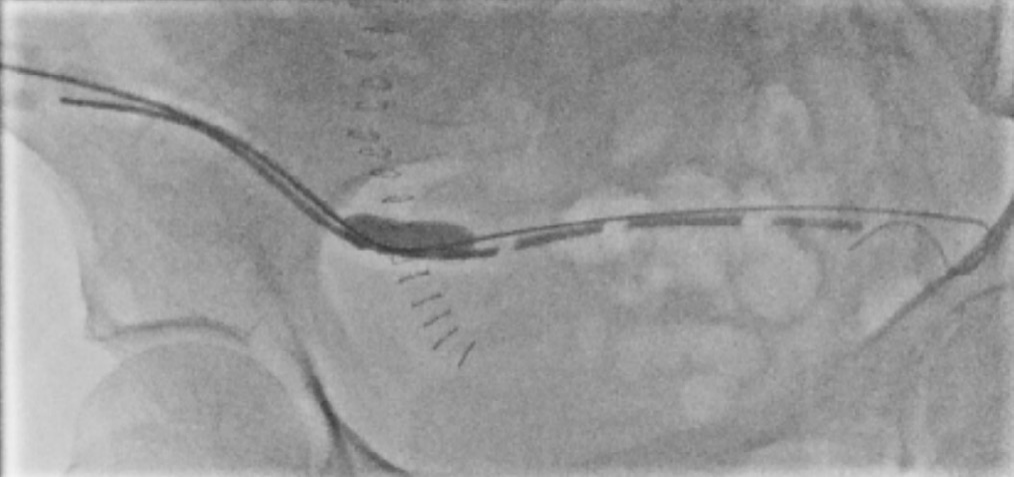
Resolution of intraluminal waisting upon focal dilatation

Consequently, a 180 cm 0.035-inch Amplatz support wire (cook medical, Indiana, United States) was advanced through the drain lumen under fluoroscopic guidance. A 9 × 40 mm vascular Charger balloon (Boston Scientific, Massachusetts, United States) was advanced over the wire. Serial dilatations were performed along the length of the drain with the anticipation of releasing the drain from any potential adhesions. Waisting was identified within the drain lumen (Figure 3). Further focal balloon dilatation was performed under recommended pressures according to manufacturer guidelines, resolving the intraluminal waisting (Figure 4). The drain was gently withdrawn over the wire with minimal effort or resistance. *Ex-vivo* inspection of the drain showed no evidence of damage and fluoroscopic imaging confirmed no retained fragments. The patient made an uneventful recovery following the procedure and was discharged one week later with no further interventions required.

## Discussion

Fibrin sheath formation is a well-recognised complication of haemodialysis catheters due to repetitive trauma to the venous intima.^3^ Whilst the most effective solution is over the wire catheter replacement, this can lead to scarring, venous stenosis and infection. Inflating the tunnelled line with an angioplasty balloon over an intraluminal wire is an efficient salvage treatment method to prolong the lifespan of the line and prevent further invasive intervention.

This minimally invasive vascular technique can be adopted to assist in managing complications in various lines and drains. Balloon thrombectomy catheters have been used to treat thromboembolic disease for many years.^4^ Balloon angioplasty has also been used to assist in intravascular foreign body removal and manipulate intravascular stents.^5^

The insertion of prophylactic intra-abdominal drains to reduce post-operative surgical complications has been common practice in abdominal surgery for decades.^6^ Tethered and retained drains can lead to serious complications such as sepsis, abscess formation, adhesions and intestinal obstruction, which may require further surgical intervention.^2^

Manual and weighted traction as a technique to remove tethered drains, has been well described in the literature.^7^ An angioplasty balloon catheter has been previously used to retrieve a retained surgical drain fragment.^8^ Balloon catheters have, therefore, been implemented to remove intra-abdominal foreign bodies but to our knowledge, this method has not been employed to resolve tethered or blocked intra-abdominal drains.

## Conclusion

The described method of intraluminal catheter plasty proved to be safe and effective in removing a tethered 24 Fr Robinson’s surgical drain secondary to fibrin sheath formation, following failed conventional methods.

## Learning points

1. Intraluminal catheter plasty is a well-recognised technique for the treatment of adhered central venous catheters. However, there is little in the literature describing the role of IR in assisting the removal of large surgical drains.
2. Retained surgical drains can lead to significant complications including gastrointestinal fistulae, abscess formation and intestinal obstruction.
3. We describe a safe and effective novel technique of intraluminal catheter plasty for serial *in-situ* dilatation, to remove a tethered surgical drain following failed conventional methods.
